# A technology-enriched approach to increasing rehabilitation dose after stroke: Clinical feasibility study

**DOI:** 10.1177/02692155251333542

**Published:** 2025-04-18

**Authors:** Gillian Sweeney, Fiona Boyd, Maisie Keogh, Patrycja Lyczba, Elaine Forrest, Philip Rowe, Mark Barber, Andy Kerr

**Affiliations:** 1 South Lanarkshire Stroke and Neuro Team, Udston Hospital, NHS Lanarkshire, Glasgow, UK; 2 Department of Biomedical Engineering, 3527 University of Strathclyde, Glasgow, UK; 3 Stroke Unit, University Hospital Wishaw, Wishaw, UK; 4 Department of Medicine for the Elderly Monklands Hospital, 3077 NHS Lanarkshire, Glasgow, UK

**Keywords:** Stroke rehabilitation, multi-technology, enrichment, acute hospital stroke unit, feasibility, dose

## Abstract

**Objective:**

To assess the feasibility of a multi-technology, group based, approach to increasing rehabilitation dose early after stroke.

**Methods:**

Mixed methods design reporting recruitment, dropout, safety, dose and acceptability.

**Setting:**

Acute Hospital Stroke Unit

**Participants:**

Sixty stroke patients, 9.0 median (IQR 12.8) days after stroke, referred for rehabilitation, without contraindications to light exercise.

**Intervention:**

Personalised rehabilitation delivered in supervised groups, using a multi-technology rehabilitation gym, in addition to usual care.

**Main measures:**

Feasibility was based on achieving recruitment rates over 3.2 per month, dropout rates below 6%, absence of suspected unexpected serious adverse reactions and shoulder pain prevalence below 60%. Acceptability was derived from interviews with the clinical team. Dose (rehabilitation time) was recorded manually. Function was measured with the modified Rivermead Mobility Index and Therapy Outcome Measure.

**Results:**

Feasibility was satisfactory with high recruitment rates (6 per month), low dropout (2%), no suspected unexpected serious adverse reactions and low prevalence (19%) of shoulder pain. Thematic analysis of interview data indicated the clinical team (n = 9) found the intervention acceptable and identified organisational constraints to higher doses. Participants attended an average of 9.1 (1–32) sessions during their hospital stay (23.0 days, SD 19.7), with sessions lasting 52 min (SD 15.7), on average. The modified Rivermead Mobility Index and Therapy Outcome Measure increased by 17.9 (SD 8.6) and 5.7 points (SD 2.4), respectively.

**Conclusions:**

Strong feasibility findings support future trials of multi-technology, group-based rehabilitation. This novel approach is an encouraging step toward achieving recommended doses of rehabilitation after stroke but needs further investigation.

## Introduction

One in three people, globally, live with a health condition that could benefit from rehabilitation.^1^ Meeting this overwhelming demand is beyond the reach of most, if not all, public health services, not least because of the insufficient workforce.^2,3^ The current, evidence-based, guidelines for rehabilitation after stroke in the UK and Ireland^4^ recommend intensive rehabilitation with a minimum of 3 hours a day of multi-disciplinary rehabilitation. This recommendation is based on good evidence of efficacy,^5–9^ despite methodological limitations, but faces implementation challenges given the historical inability to meet previous, more modest, guidelines.^10^ For many people, this will mean receiving either suboptimal rehabilitation or no rehabilitation at all, potentially limiting recovery and leading to higher future health, social care and societal costs.

Technological interventions have reached the point of maturity where they could help meet this need, in a personalised and equitable way. Rehabilitation technology such as power-assisted exercise equipment, virtual reality, electrical stimulation, digital health apps and adapted treadmills have evidence of effect across a range of conditions; for example stroke^11^ and Parkinson's Disease^12^ as well as age-related disability.^13^ This technology is, however, underutilised with access described as poor, or non-existent, in the public sector of many countries, including the UK, with several barriers to adoption reported including training, technical skills, knowledge and limited space.^14,15^ Furthermore, training with technology is typically offered for a single impairment when combinations of impairments (motor, communication and cognitive) are experienced by stroke survivors.

A technology-enriched rehabilitation gym has been developed in partnership with a chronic stroke population using a participatory model of Co-Creation.^16^ This novel technological intervention combines commercially available motor, communication and cognitive training technology which have been selected by users and rehabilitation professionals for their usability and adherence, and based on published data of effect and our own observations.^17^ An individual programme, designed by a health professional around personal goals and standard outcome measures, uses the selected technology, in small groups, with supervision and assistance provided by appropriately trained individuals.

The promising changes in function we have observed with this approach, in a chronic population (39.0 months (SD 29.2) post-stroke),^17^ may be greater if applied early after stroke when neuroplastic mechanisms are considered optimal,^18,19^ although the evidence for this remains largely limited to animal models.^20^

Our hypothesis is that this group based, combined technological intervention, can be successfully delivered on an acute stroke unit, albeit with some cultural and organisational challenges, to increase the rehabilitation dose. The aim of this study was, therefore, to test the feasibility (recruitment, dropout, safety, dose and staff acceptance) of a technology-enriched approach to increasing rehabilitation dose in sub-acute stroke in-patients.

## Methods

The study had a mixed methods approach to assessing the feasibility and acceptability of technology-enriched rehabilitation with stroke in-patients in an NHS primary stroke centre. This research approach follows Medical Research Council guidelines for developing complex interventions model.^18^

Patients referred for rehabilitation following a diagnosis of stroke were invited to participate. Patients with contraindications to light exercise were excluded. Table 1 details the main eligibility criteria. The study protocol was approved by the local ethics committee (23/SS/0098) and registered with ClinicalTrials.gov (NCT05981729).

**Table 1.** Eligibility criteria for study.

Inclusion criteria	Exclusion criteria
Diagnosis of new stroke by NHS stroke physician	Acutely medically unwell
More than 48 h since stroke event	Active cardiac disease, such as unstable angina
Deemed medically fit for rehabilitation	Active delirium/significant levels of confusion
Deemed to require rehabilitation	Seizure within past 7 days
Able to provide informed consent	Individual currently being managed under the Adults with Incapacity Act, unless the responsible medic has noted within the document that the individual has capacity to consent to rehabilitation research^a^
	Known pregnancy

a This criterion was added 3 months into the study with the decision to invite participation left to the clinical judgement of the patient's Medical Consultant. The amendment was approved by the Ethics Committee.

Following informed consent, participants attended the technology-enriched rehabilitation gym, which was located on the acute stroke unit, for an initial induction and goal setting session. The gym consisted of a range of rehabilitation technology (see Table 2 for full list) which have been approved for use and tested with chronic stroke participants.^17^

**Table 2.** Equipment available in the technology-enriched rehabilitation gym.

Equipment	Purpose	Model and manufacturer	Comments
VR-adapted treadmill	Gait and balance training	N-Mill, Motek Medical, NL	Used bespoke optical tracking system
Large, height adjustable, computer tablet with digital health apps	Communication and cognitive training performed in standing or sitting	Tiny Tablet, UK	Apps included TACTUS communication and attention apps
GripAble hand trainer	Upper limb training and cognitive training	GripAble, UK	
Mirror therapy	Mirror therapy Priming & upper limb training	Bespoke	Required use of a customised manual
Seated rower (Power-assisted exercise equipment)	General exercise, aerobic, strength, flexibility	Innerva, UK	
Cross cycle trainer (Power-assisted exercise equipment)	General exercise, aerobic, strength, flexibility	Innerva, UK	
Seated climber (Power-assisted exercise equipment)	General exercise, aerobic, strength, flexibility	Innerva, UK	
Power-assisted bike	General exercise, aerobic, strength, flexibility	RECK, DE	
VR headset	Upper limb and balance	Meta Quest headset, USA, using software designed by Incisiv, UK	Included bespoke software targeting upper limb co-ordination and balance

Following an initial induction session with one of the research team (GS/FB/MK) and/or supervising members of the clinical team, participants were offered daily sessions, in groups of up to six individuals, every weekday until discharged from hospital. Each session lasted a maximum of 2 hours and was structured according to their personal goals and in consultation with the clinical team. Two members of the clinical team (rehabilitation assistants, band 4 and 2) provided supervision, this included physical assistance with some of the technology (e.g. the seated cross trainer) and help operating the technology, if required, but did not provide conventional hands-on therapy and encouraged individuals to use the technology on their own where appropriate. These sessions were in addition to participant's ongoing rehabilitation provided by the clinical team, including physiotherapy, occupational therapy and speech therapy. The details of usual rehabilitation (frequency, content and duration) were not fully available to the research team.

The feasibility of this technological rehabilitation intervention, in a sub-acute stroke population, was assessed by specific criteria. Recruitment rate (numbers consenting per month) should reach 3.2 and dropout (percentage not completing study) should be below 6%; these are the upper quartile and median values, respectively, reported by McGill et al. in a systematic review of stroke rehabilitation trials.^19^ Safety was assessed from reported adverse events, in particular the absence of any suspected unexpected serious adverse reactions, and a prevalence of hemiplegic shoulder pain that did not exceed the 60%, as reported by Li et al.^21^ Dosage was calculated as the total number of minutes attended per person and the average duration of daily sessions. These data were tested for normality with the Anderson Darling test and described with appropriate average and variability statistics.

To understand whether the intervention was acceptable to the clinical team, and gain insight for a future multi-centre trial, all staff directly involved in patient care were invited by email to participate in semi-structured interviews. Following an informed consent process, nine members of staff (one physician, one nurse and seven therapists) agreed to be interviewed (semi-structured) by one of the research team (MK or FB) – see supplementary file for the interview schedule. Once the interviews were transcribed, a thematic analysis approach was undertaken by a third researcher, not involved in the interviews (GS), to define and explore key themes,^22^ these findings were corroborated with the interviewing researchers to ensure important information such as tone and non-verbal cues were not missed.

Finally, standard outcome measures of function (Modified Rivermead Mobility Index and the Therapy Outcome Measure) were used to characterise participants’ functional ability before starting and track any change that had occurred by discharge. Given the lack of control group, we did not infer any change to the intervention; the data are simply presented for context and to allow sample size calculations for future studies.

## Results

**Figure 1.**
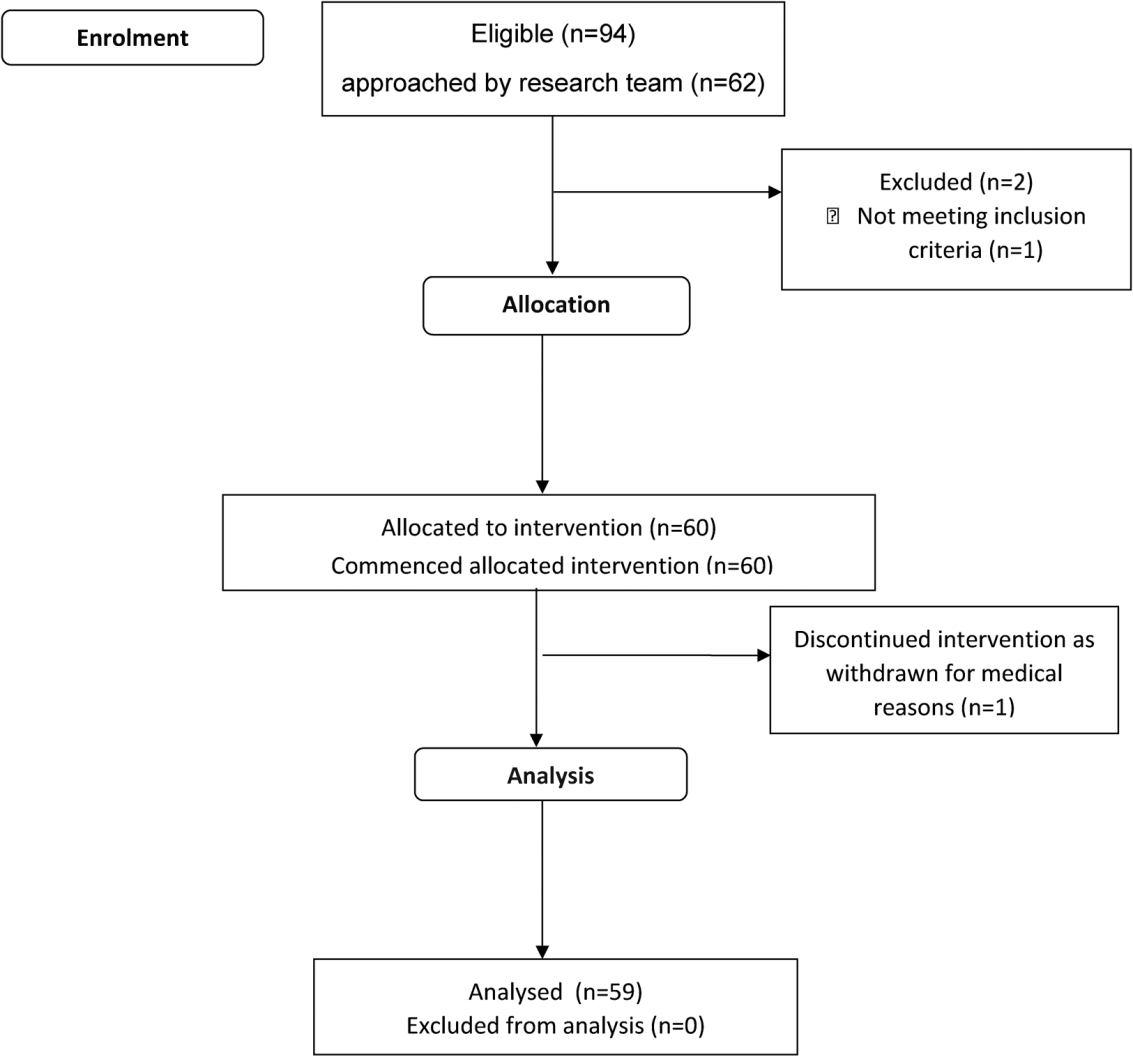
Consort flow diagram of recruitment.

During a 10-month recruitment period, 94 patients were considered eligible for the study, 62 were approached by the research team, 61 met the inclusion criteria and 60 consented, giving a recruitment rate of 6 per month. Reasons for not approaching 32/94 of eligible participants included restricted access to the research team (for consent and induction) and a 6-week period of suspension waiting for an amendment to be approved by the ethics committee. Participants were 69.6 (14.0) years old, 33 (55%) were male, 49 had an ischaemic and 11 had a haemorrhagic stroke. Participants were all referred for rehabilitation with either right sided (22/60, 37%), left sided (27/60, 45%) or bilateral (11/60, 18%) weakness. The majority (41/60, 68%) required some equipment to help with transfers which is consistent with their relatively low function (average Modified Rivermead Mobility Index of 7.0 (13.0) out of a maximum of 40), see Table 3 for details. One participant dropped out as a medical precaution producing a dropout rate of 1/60 (2%). Participants were enrolled in the study for an average of 23.0 days (SD 19.7), ending when they were discharged from hospital. See Figure 1 for consort flowchart and Table 5 for further details on participation.

**Table 3.** Participant characteristics at recruitment, n = 60.

	Mean (SD) or Median (IQR)*	Range
Age (years)	69.6 (14.0)	36–94
Gender (Male/Female)	33/27	
Days since stroke	9.0 (12.8)*	2–190
Stroke type	Ischaemic 49, Haemorrhagic 11	
Side affected	Right 22, Left 27, Both 11	
Requiring transfer equipment	41/60	
Modified Rivermead Mobility Index	7.0 (13.0)*	3.0–31.0
Shoulder pain prevalence	9/60 (15%)	
Aphasia	8/60 (13%)	

There were no suspected unexpected serious adverse reactions to the intervention, five individuals reported mild joint and/or muscle pain, which resolved with 2 days without medical intervention. The prevalence of hemiplegic shoulder pain was 15% before the intervention, five new cases of hemiplegic shoulder pain (8%) were reported during the intervention and three (5%) cases resolved during the intervention giving a prevalence of 19%, below the 60% reported by Li et al.,^21^ see Table 4.

**Table 4.** Safety, adverse events and shoulder pain prevalence data.

Adverse event	Number	Days to resolve	Comment
Joint and muscle pain	5/59 (8%)	2 days	No medical intervention required
New shoulder pain	5/59 (8%)	Ongoing monitoring	No medical intervention required

Participants (n = 60) were enrolled in the study for an average of 23.0 (19.7) days, completing 9.1 (7.4) sessions with an average duration of 52.0 min, ranging from 21.7 to 93.3. The greatest percentage of time was spent using the power-assisted technology, which all participants used, bar one, activity with the other equipment varied according to need, see Tables 5 and 6.

**Table 5.** Rehabilitation dose, n = 60.

	Mean (SD)	Range
Number of days enrolled in study; Recruitment until hospital discharge	23.0 (19.7)	2–81
Total sessions attended	9.1 (7.4)	1–32
Average duration of sessions (mins)	52.0 (15.7)	21.7–93.3
Total rehab time per participant (mins)	508.4 (521.2)	40.0–2567.0

**Table 6.** Average (median) time spent training with individual rehabilitation technologies.

	Median (IQR)	Percentage of total engagement (SD)	Range
Power-assisted exercise, n = 59	150.0 (185.0)	44.6% (23.7)	5–950
Cognitive training apps, n = 44	70.0 (163.8)	21.6% (14.9)	10–1271
Upper limb (e.g. GripAble, VR and mirror therapy), n = 35	100.0 (175.0)	26.6% (17.2)	10–940
Treadmill/balance, n = 13	40.0 (62.5)	6.2% (3.7)	10–140
Communication apps, n = 6	100.0 (367.0)	49.5% (30.0)	40–870

The majority (86.6%) of sessions were supervised by assistant rehabilitation practitioners (NHS bands 4 and 2), see Table 7.

**Table 7.** Staff supervision.

Total sessions	Sessions supervised by assistant rehabilitation practitioners	Sessions requiring registered staff	Number of participants per session
142	123 (86.6%)	19 (13.4%)	3.45 (1.29)

There were substantial changes in the functional ability of participants with a 17.9 (8.6) point improvement in the Modified Rivermead Mobility Index and 5.7 on the Therapy Outcome Measure, although these was only available from 58/59 and 32/59 of completing participants, see Table 8.

**Table 8.** Functional outcome measures at recruitment, discharge and change.

Outcome measure	Recruitment	Discharge	Change
Modified Rivermead Mobility Index (median/IQR), n = 58	11.2 (8.0)	30.0 (9.5)	17.9 (8.6)
Therapy Outcome Measure (mean/SD), n = 32	8.9 (4.0)	12.9 (2.8)	5.7 (3.4)

Two clear themes emerged from staff interviews. These were 1) benefits of attending, for both patients and staff and 2) barriers to attending which were either personal to patients or organisational (ward and research team). These are described below and have been condensed in Table 9.

**Table 9.** Themes and sub-themes identified from the staff interviews.

Theme	Sub-themes	Supporting example quotes
Benefits	Staff benefits	‘takes a bit of pressure off the staff down in the ward’. ‘what they seem to be achieving from it, is a benefit to the service as a whole’
	Patient benefits	‘the increased input in terms of (rehabilitation) time, and what the patients are getting’. ‘It's physically good for them and also mentally I believe that being able to go to this different area of the hospital to do something different for themselves, this is helping them this is they're not sitting and doing nothing at the side of the bed’
Barriers	Personal	‘they can be a little bit not knowing quite what to expect’, ‘patients that are struggling with fatigue sometimes only manage an extra 20 minutes, but others manage for the whole 2 hours’
	Organisational	‘more regular meetings and frequent input from the research team would have helped identify and pick patients up quicker’ ‘the challenge is maintaining the staff, training the staff, and making sure that there's enough staff to allow the patients to participate and to supervise them when necessary.’

### Benefits of attending

All staff commented positively about the multi-technology-enriched rehabilitation sessions. They felt that enabling participants to increase rehabilitation intensity had impacted on confidence, mood, and ability to engage in self-practice. Staff also commented that pressure on ward staff had been eased as a consequence of patients attending group sessions.

### Barriers to attending

Some staff (n = 4) felt that patients may have been put off by unfamiliarity with the technology and others (n = 2) cited post-stroke fatigue as a possible barrier to engagement. Staff supervising the sessions also mentioned that attendance was impacted by visitors and medical/nursing procedures. All interviewed staff, however, felt these barriers could be overcome with support and flexibility delivering the sessions (e.g. ‘..once they've come in and seen the layout and what's available, then they're a bit more comfortable and confident at taking part in it’).

For future research studies, staff felt greater availability of the research team would improve recruitment of patients who were in for a shorter stay and placed importance on ensuring aphasia friendly resources were available.

Interviews were also conducted with participants and carers; the analysis from these interviews is being prepared for a future publication that will focus on the patient experience.

## Discussion

Co-created with stroke survivors and rehabilitation professionals this multi-technology, group based, rehabilitation intervention was assessed for feasibility with sub-acute stroke patients (>48 h post-diagnosis until hospital discharge) and acceptability by the clinical staff in an NHS primary stroke centre. The high recruitment (6 per month) and low dropout (2%) rates, combined with the low incidence and minor nature of safety events, strongly support the feasibility of this intervention for future efficacy studies early after stroke. Furthermore, the overwhelmingly positive reception from the clinical team, with a perceived impact on patient mood and motivation, as well as ward efficiency, suggests the intervention is not only clinically acceptable but also desirable.

These feasibility outcomes outperform findings from a systematic review of recruitment to stroke rehabilitation trials (n = 512) that reported median recruitment rates of 1.5 per month and dropout rates of 6%.^19^ These encouraging outcomes should nevertheless be tested at other sites which may have less staff resource or have additional barriers to attendance, for example in community rehabilitation sites.

Importantly the intervention delivered an additional 52 min, on average, of daily rehabilitation during the in-patient stay of sub-acute stroke patients, supporting our hypothesis. While this increase is a substantial improvement on previous reports,^10^ it remains below the recommended levels.^4^ Data from the staff interviews offered some insight on reasons for this, along with potential solutions. Two patient-centred factors were identified as reasons for missing sessions: 1) fatigue and 2) being available for visitors. In addition, there were organisational limiting factors including staff availability (two staff were required to be present in the gym at all times for safety) and availability for medical procedures (e.g. MRI scans). These constraints on access to rehabilitation have been reported before,^10^ are largely organisational and could be resolved (at least partially) with changes to the ward operation; for example protecting a daily 2-h rehab period and opening access to the facility at weekends and evenings. Understandably, medical investigations/procedures will always take priority in an acute hospital setting but there may be compromises and organisational change that enable greater rehabilitation engagement.

Post-stroke fatigue on the other hand is less easy to resolve and presents a major barrier to effective rehabilitation.^23^ Possible strategies include pharmacological^24^ and non-pharmacological interventions such as cognitive behavioural therapy and neurostimulation.^25^ The outcomes from many of these interventions are, however, variable and generally disappointing with post-stroke fatigue continuing to have a high prevalence many years after stroke. Paradoxically, exercise itself may improve post-stroke fatigue^25^ with the challenge then to motivate and then maintain active participation. Rehabilitation games could be used more effectively to provide this motivation^26^ but need to be co-created with users to improve long-term engagement.

These promising findings should be considered in context of the study limitations which included not approaching all eligible patients to participate and not recording the usual rehabilitation dose, which may have been affected by the intervention. Further research is recommended to address these limitations and add additional measurements to determine both the clinical and cost-effectiveness to enable the development of a sustainable, evidence-based intervention that can be translated to a range of clinical and non-clinical sites.

While acknowledging these limitations, it is worth highlighting that our hypothesis was supported. A technology-based approach to rehabilitation was feasible and acceptable on a primary stroke centre and delivered almost 1 h of additional rehabilitation. This was achieved in the context of historically very low doses of rehabilitation being provided during the first 3 months after stroke with an average of 12.1 (16.8) total physiotherapy sessions.^27^ The challenge in stroke rehabilitation is to find a model that can practically, equitably and sustainably, meet the guideline NICE recommendations.^28^ Supervised group sessions in a technology-enriched gym offers a feasible, clinically acceptable, method for increasing rehabilitation dose that can be widely adopted in state managed health services. The intervention increased the volume of daily rehabilitation, exceeding previous guidelines, and may, with some organisational adjustments and increased familiarity, achieve current guideline recommendations.

**Clinical messages**

- A multi-technology, group based, rehabilitation intervention is feasible in sub-acute stroke patients.
- A multi-technology, group based, rehabilitation intervention is acceptable to the clinical team on an NHS primary stroke centre.
- A multi-technology, group based, rehabilitation intervention can provide 52 min of additional rehabilitation, per day.
- This additional rehabilitation did not raise the incidence of adverse events, including hemiplegic shoulder pain.

## Supplemental Material

sj-docx-1-cre-10.1177_02692155251333542 - Supplemental material for A technology-enriched approach to increasing rehabilitation dose after stroke: Clinical feasibility study Supplemental material, sj-docx-1-cre-10.1177_02692155251333542 for A technology-enriched approach to increasing rehabilitation dose after stroke: Clinical feasibility study by Gillian Sweeney, Fiona Boyd, Maisie Keogh, Patrycja Lyczba, Elaine Forrest, Philip Rowe, Mark Barber and Andy Kerr in Clinical Rehabilitation
