# Supplementary material for: A technology-enriched approach to increasing rehabilitation dose after stroke: Clinical feasibility study
Source: Clin Rehabil. 2025 Apr 18;39(6):740–9. doi: 10.1177/02692155251333542 (PMC12141766; doi:10.1177/02692155251333542)
Supplement: sj-docx-1-cre-10.1177_02692155251333542 - Supplemental material for A technology-enriched approach to increasing rehabilitation dose after stroke: Clinical feasibility study [file sj-docx-1-cre-10.1177_02692155251333542.docx]

# Semi-structured interview schedule

# NHS staff interviews

| **Themes** | **Questions** |
| --- | --- |
| Prior Perceptions and experience of technology | 1. Before this study began, what experience did you have in using technology in rehabilitation?   If yes; what have you used before?  If no; What do you think has prevented you from using?   1. What do you understand about the use of technology in rehabilitation? 2. Did you have any concerns about using rehabilitation technology? |
| Training | 1. Can you tell me about your involvement with the study/ what rehabilitation technology have you had the opportunity to use with participants? 2. What form of training did you receive in the use of rehabilitation technology? (Prompt; was this enough?) 3. How confident do you feel now about (as appropriate)   prescribing/demonstrating/supporting participants with rehabilitation technology?   1. Is there anything that would help you to feel more confident? |
| Perspective using with patients/participants | 1. Do you feel there are any benefits to **patients** in using rehabilitation technology? 2. Do you feel there are any challenges to **patients** in using rehabilitation technology? 3. Do you feel rehabilitation technology offers any benefits to **staff and the service**? 4. Do you feel rehabilitation technology presents any challenges to **staff and the service**? |
| Service Provision | 1. Do you feel the use of rehabilitation technology has the potential to affect your service’s ability to meet national clinical guidelines around rehabilitation? 2. Can you tell me about whether you feel the use of rehabilitation technology could be potentially useful at other stages of the rehabilitation journey (such as when discharged from hospital)? |
| Overall perceptions and opinions | 1. Can you describe give a summary of your experience during this study? 2. Is there anything that you could suggest to make similar studies better in the future? 3. Is there anything you would like to add that we haven’t already discussed today? |
